# Dunbar syndrome - case report

**DOI:** 10.1590/1677-5449.202300301

**Published:** 2023-07-17

**Authors:** Sarah Maria Lemos de Campos, Rafael Prado Pessoa, João Paulo de Araújo Pelegrini, Henrique Fernandes Silveira, Maria Fernanda Lopes Diniz, Maria Passos Bianchini, Leonardo Soares Lopes, Marcus Eduardo Valadares Meireles Martins da Costa

**Affiliations:** 1 Hospital Mater Dei Unidade Santo Agostinho, Belo Horizonte, MG, Brasil.

**Keywords:** ligament, Dunbar, celiac trunk

## Abstract

Dunbar syndrome is diagnosed by excluding other possible causes of abdominal pains. Surgical treatment comprises complete dissection of the ligament and the surrounding nerve ganglion. This report describes the case of a previously healthy 45-year-old male patient who presented with epigastric abdominal pain irradiating to the back and weakness. Initially, abdominal computed tomography was ordered, showing arteriopathy of the celiac trunk and mesenteric artery with stenosis. The patient underwent surgical treatment because of the refractory pain, but findings were nonspecific. It was necessary to continue workup with serial angiotomography to follow the case. After around 6 months, thickening of the arcuate ligament was found, with compression of the proximal third of the celiac trunk and 80% stenosis. The patient therefore underwent laparoscopy to relieve celiac trunk compression, with satisfactory postoperative recovery.

## INTRODUCTION

Dunbar syndrome (DS), also known as celiac artery compression syndrome or median arcuate ligament syndrome, is a rare disease characterized by chronic and recurrent abdominal pains related to compression of the celiac trunk by the median arcuate ligament.^1^ Symptoms linked to this syndrome include the triad of postprandial abdominal pains (because of visceral ischemia caused by compression of the celiac artery), weight loss, and abdominal murmur and there may also be vomiting, nausea, and diarrhea.^2,3^

This report describes the case of a patient diagnosed with DS. The study was approved by the Research Ethics Committee at our institution (decision number 5.719.672), as mandated by the CARE guideline.^4^

## CASE DESCRIPTION

The patient was a previously healthy 45-year-old male who had never undergone surgery before and sought hospital care for intense epigastric abdominal pains irradiating to the back and weakness. The possibility of acute appendicitis was ventured during the consultation, because of involuntary guarding at the iliac fossa during physical examination and leukocytosis. It was decided to supplement work up with abdominal computed tomography (CT). The CT report described arteriopathy of the celiac trunk, with caliber reduced by approximately 73%, and arteriopathy of the superior mesenteric artery, with 40% narrowing; enlarged retroperitoneal lymph nodes; and thickened gallbladder. The patient was scheduled for exploratory laparoscopy in view of the refractory nature of his pain. No abnormalities that could explain the patient’s pain were identified during this procedure and all possible causes of acute abdomen were ruled out. The patient was discharged from hospital promptly, with improved symptoms.

**Figure 1:**
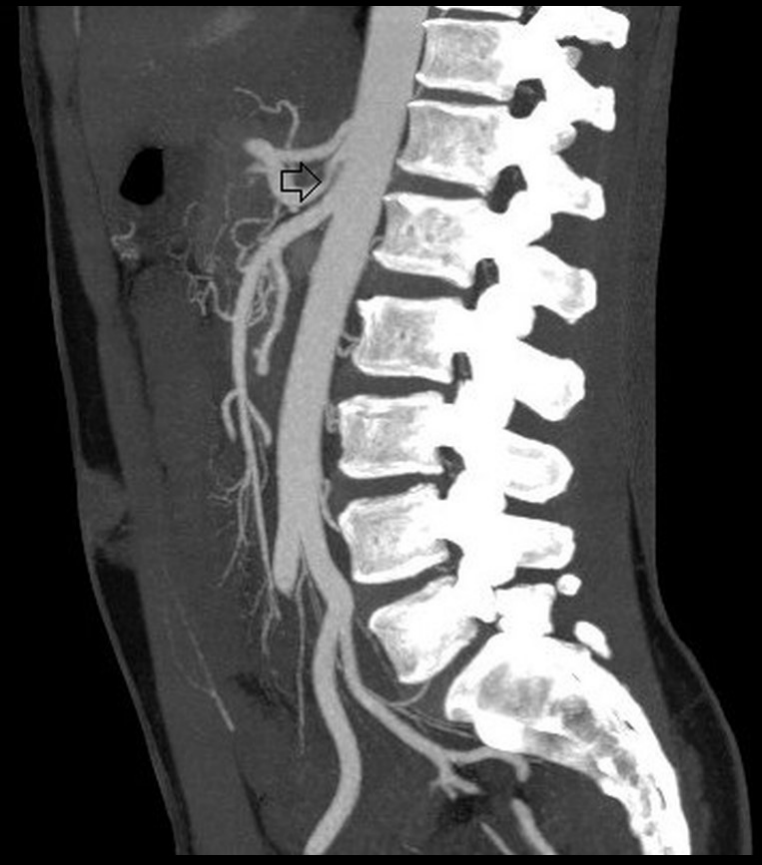
Sagittal reconstruction angiotomography of the abdominal aorta and its branches. The arrow indicates preoperative stenosis of the proximal portion of the celiac trunk and the post-stenotic dilatation.

After the tomographic findings from the previous admission, the patient was scheduled for abdominal angiotomography the following month, which found stenosis of the celiac trunk (50%) (Figure 1). After around 6 months, another angiotomography study was performed, which is when thickening of the median arcuate ligament of the diaphragm was observed for the first time, with compression and 80% stenosis of the proximal third of the celiac trunk.

**Figure 2:**
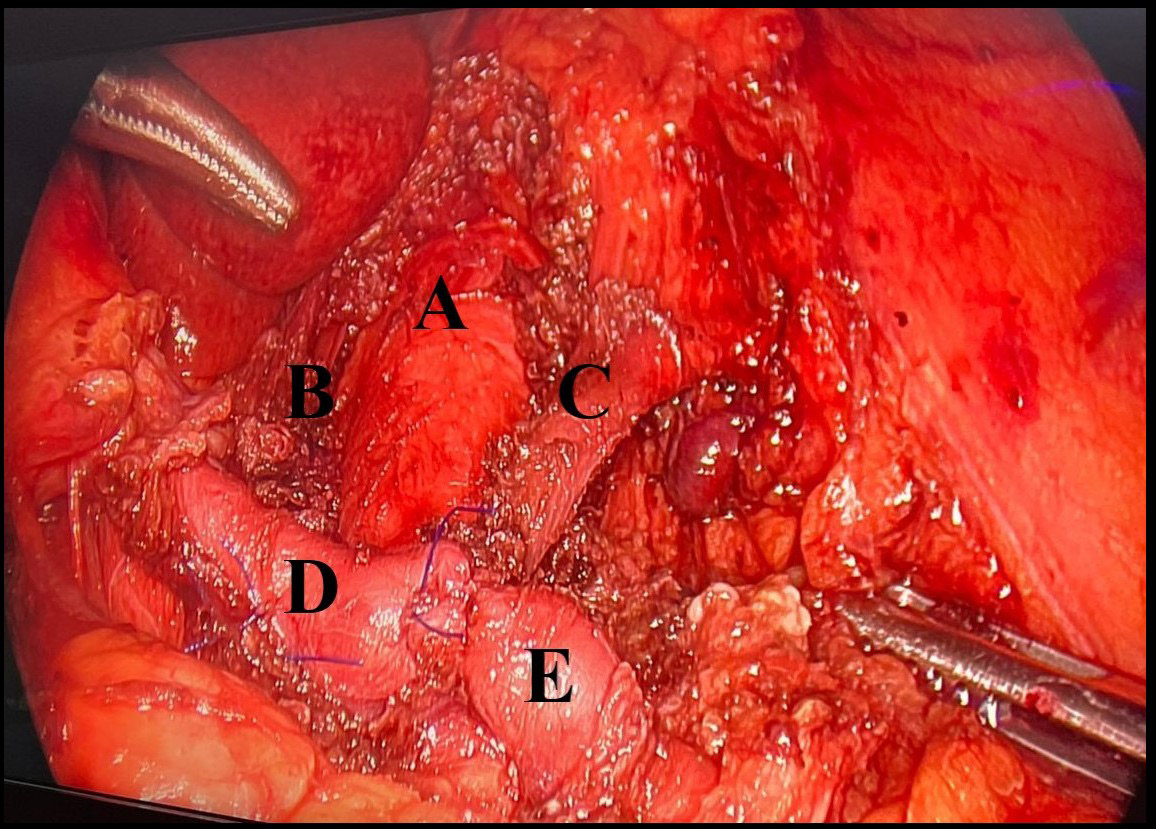
Intraoperative image showing the celiac trunk after section of the fibers of the arcuate ligament. (A) Abdominal aorta; (B) Fibers of the arcuate ligament; (C) Left gastric artery; (D) Common hepatic artery with primary arteriorrhaphy; (E) Splenic artery with primary arteriorrhaphy.

The patient underwent laparoscopic surgery to relieve compression of the celiac trunk. During the operation, the common hepatic artery was dissected in the direction of the celiac trunk, where the fibers of the arcuate ligament were released and the adjacent celiac ganglion was removed. While releasing the celiac trunk, it was necessary to partially section the splenic and common hepatic arteries to gain access to the fibers of the arcuate ligament, since these vessels were intrinsically linked to the filaments, which caused moderate bleeding, that was controlled with primary arteriorrhaphy (Figure 2). The patient was given intraoperative blood transfusion with packed red blood cells and was taken to intensive care for postoperative recovery, progressing to hospital discharge 3 days later.

**Figure 3:**
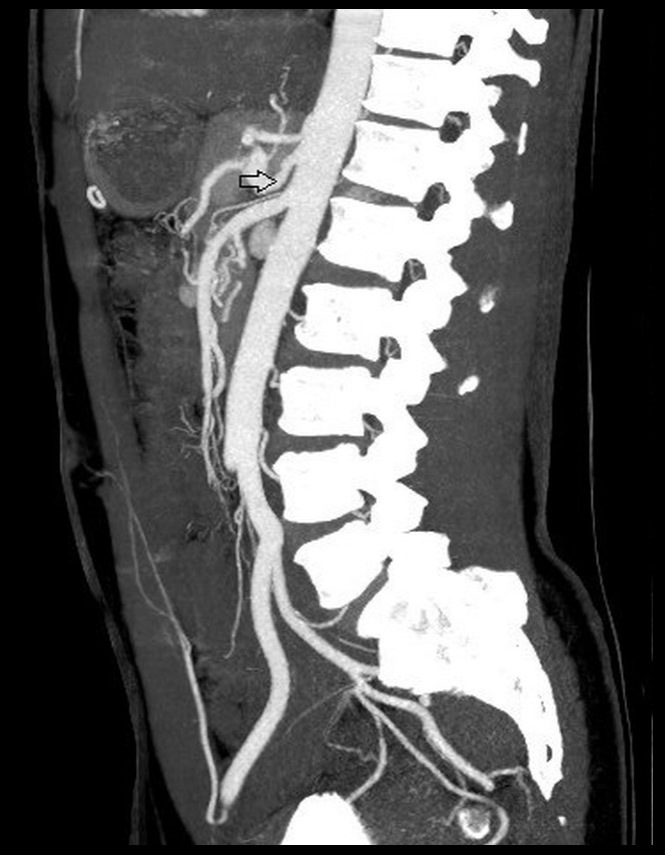
Sagittal reconstruction angiotomography of the abdominal aorta and its branches. The arrow indicates the improved celiac trunk stenosis after surgical intervention.

The patient remained asymptomatic during the postoperative period and underwent a control angiotomography (Figure 3) 2 months after surgery, which showed improved celiac trunk stenosis at 60-70%, in addition to critical stenosis of almost 100% of the proximal segment of the splenic artery, with distal flow fed via collateral circulation originating from the gastroduodenal artery. Currently, 1 year after the surgical intervention, the patient remains asymptomatic and has chosen not to undergo further imaging exams, in view of his stable condition.

## DISCUSSION

Dunbar syndrome was identified by Harjola in 1963, who described the case of a patient with postprandial abdominal pains and a murmur in the epigastric area.^5^ After reporting a series of cases in 1965, Dunbar described the constellation of signs and symptoms of postprandial abdominal pains, weight loss, vomiting, diarrhea, and abdominal murmur and related them to compression of the celiac trunk by the median arcuate ligament.^3^ The incidence of DS in the population is not fully known, but the syndrome is more common in women than in men (4:1), at ages from 30 to 50 years, and in patients with a lean biotype.^6^

The physiopathogenesis of DS has not been entirely elucidated, but it is based on two primary mechanisms: ischemia and neuropathy, either one of which may predominate.^7^ The ischemic mechanism is caused by the intimate relationship between the median arcuate ligament and the celiac trunk. The median arcuate ligament is a band of fibrous tendon that connects the left and right crura of the diaphragm, forming the anterior border of the aortic hiatus.^3,6^ Both of the diaphragm crura originate at the vertebral bodies of the first to fourth lumbar vertebra. The celiac trunk originates from the abdominal aorta, at a level between the eleventh thoracic vertebra and the first lumbar vertebra. As a result, people who have a higher celiac trunk origin or a lower median arcuate ligament insertion have a greater predisposition to developing extrinsic compression of the celiac trunk, giving rise to the ischemic mechanism of DS.^7^

In turn, the neuropathic mechanism can be caused by the position of the celiac plexus or celiac ganglion. The celiac plexus is found adjacent to the median arcuate ligament and originates from the preganglionic splanchnic nerves, the somatic branches of the phrenic and vagus nerves, the preganglionic parasympathetic nerves, and the postganglionic sympathetic fibers.^1,8^ The pain associated with compression of the celiac trunk may be mediated by this plexus, since patients given a local anesthetic blockade of the celiac plexus exhibit improvement of symptoms.^8^

Diagnosis of DS starts with a clinical suspicion in patients who present with signs and symptoms of the syndrome, but must be confirmed with imaging exams.^9^ The imaging exams most often used are angiography, ultrasound with Doppler, tomography/angiotomography, and magnetic resonance imaging/angiography.^9,10^ Angiography is considered the gold standard for diagnosis of DS.^5^ However, with the development of multislice angiotomography, it has become possible to obtain very accurate images, with good resolution and a high rate of detection of lesions with a much less invasive technique than arteriography. Angiotomography is thus an excellent choice for diagnosing DS.^11^

Arteriographic findings of compression of the celiac trunk, particularly in images acquired in lateral projection, have been described as typical, showing stenosis of the proximal portion of the celiac trunk and displacement downwards in the direction of the aorta, followed by a cranial tilt, forming a U or J shape, which has also been called a hook-like appearance or a hook sign.^7^ With varying frequency, increased collateral arteries of the pancreaticoduodenal arcade and post-stenotic dilatation may also be observed. A combination of these signs and symptoms in the gastrointestinal tract after other causes have been ruled out appears to justify surgical decompression of the celiac trunk.^7,9^

Since we needed to rule out more common causes of abdominal pains in this case, we ordered laboratory tests and abdominal CT, which showed a reduction in the caliber of the celiac trunk. Later, we performed angiotomography, which showed thickening of the median arcuate ligament, with compression of the celiac trunk. Dunbar syndrome is diagnosed on the basis of presence of gastrointestinal symptoms in combination association with suggestive radiological signs, in the absence of other causes that could explain the patient’s symptoms.^7^ We thus arrive at a diagnosis of DS.

With regard to treatment, although angioplasty with stenting is considered a good option for treatment of the atherosclerotic component of celiac trunk obstruction, it does not offer good results if used as the only treatment for celiac artery compression, since the extrinsic compression impedes the stent from adequately expanding the vessel. Moreover, this technique is ineffective as a treatment for the nervous component of DS. Angioplasty has an important role to play as a secondary procedure in cases of residual stenosis after decompression of the celiac trunk.^11^

The first choice treatment for DS consists of surgery to decompress the celiac trunk by releasing the median arcuate ligament and removal of the celiac ganglion, in order to treat both components of the disease. Surgery can be performed via an open or laparoscopic access, with laparoscopy being preferred because it is less invasive and achieves earlier control of the symptoms. However, it is nevertheless subject to a high rate of conversion to open surgery because of the high rate of morbidity associated with acute bleeding of the suprailiac aorta and its branches.^2,11^ Refinement of the techniques, choice of an experienced surgeon, and careful identification of high-risk patients can reduce the incidence of conversion.^2^ Treating only the ischemic or the neurological component can prove effective, since they can occur in isolation.^11,12^ In the present case, the technique chosen was videolaparoscopic decompression of the celiac trunk using an ultrasonic scalpel.

## CONCLUSIONS

Diagnosis of Dunbar syndrome is still a challenge, but, as radiographic techniques improve, compression of the celiac trunk is being identified with growing frequency, facilitating diagnosis. Recent studies have shown satisfactory results with surgical decompression, whether with open or laparoscopic surgery, and the most important consideration is the surgeon’s experience with each technique. After surgical intervention, it is necessary to perform control angiotomography to monitor the postoperative reduction of celiac trunk stenosis.
