# Supplementary material for: Dunbar syndrome - case report
Source: J Vasc Bras. 2023 Jul 17;22:e20230030. doi: 10.1590/1677-5449.202300301 (PMC10421585; doi:10.1590/1677-5449.202300301)
Supplement: Translation

# Translation: Síndrome de Dunbar - relato de caso

**DOI:** 10.1590/1677-5449.202300302

**Authors:** Sarah Maria Lemos de Campos, Rafael Prado Pessoa, João Paulo de Araújo Pelegrini, Henrique Fernandes Silveira, Maria Fernanda Lopes Diniz, Maria Passos Bianchini, Leonardo Soares Lopes, Marcus Eduardo Valadares Meireles Martins da Costa

**Affiliations:** 1 Hospital Mater Dei Unidade Santo Agostinho, Belo Horizonte, MG, Brasil.

**Keywords:** ligamento, Dunbar, tronco celíaco, ligamento, Dunbar, tronco celíaco

## Abstract

A síndrome de Dunbar constitui um diagnóstico de exclusão dentro dos quadros de dor abdominal. O tratamento cirúrgico consiste na dissecção completa do ligamento e do gânglio nervoso circundante. Dessa forma, o presente caso refere-se a um paciente do sexo masculino, 45 anos, previamente hígido, com queixa de dor abdominal epigástrica com irradiação para o dorso e fraqueza. Inicialmente, foi realizada tomografia computadorizada de abdome para complementação do quadro, que evidenciou arteriopatia do tronco celíaco e da artéria mesentérica em associação com estenose. Optou-se por tratamento cirúrgico devido à refratariedade da dor, mas os achados foram inespecíficos. Houve necessidade de complementação da propedêutica com angiotomografia seriada para acompanhamento do caso. Após cerca de 6 meses, notou-se espessamento do ligamento arqueado, com compressão do terço proximal do tronco celíaco e estenose de 80%. Em meio a esse cenário, o paciente foi submetido a laparoscopia para descompressão do tronco celíaco, evoluindo satisfatoriamente no pós-operatório.

## INTRODUÇÃO

A síndrome de Dunbar (SD), também conhecida como síndrome de compressão da artéria celíaca ou síndrome do ligamento arqueado mediano, é uma doença rara caracterizada por dor abdominal crônica e recorrente relacionada à compressão do tronco celíaco pelo ligamento arqueado mediano^1^ . Os sintomas relacionados a essa síndrome incluem a tríade de dor abdominal pós-prandial (devido a isquemia visceral acarretada pela compressão da artéria celíaca), perda de peso e sopro abdominal, mas também podem incluir vômito, náusea e diarreia^2,3^ .

O presente relato descreve o caso de um paciente diagnosticado com SD. O estudo foi aprovado pelo Comitê de Ética em Pesquisa da nossa instituição (parecer número 5.719.672), conforme preconizado pela diretriz CARE^4^ .

## DESCRIÇÃO DO CASO

Paciente do sexo masculino, 45 anos, previamente hígido, sem cirurgias prévias, procurou atendimento hospitalar após apresentar quadro de dor abdominal de forte intensidade na região epigástrica com irradiação para o dorso e fraqueza. Durante a consulta, foi levantada a possibilidade de apendicite aguda devido à presença de defesa abdominal na fossa ilíaca direita durante exame físico e leucocitose. Optou-se por complementação terapêutica com tomografia computadorizada (TC) de abdome. O laudo da TC de abdome evidenciou arteriopatia do tronco celíaco, com redução do calibre em aproximadamente 73%, e da artéria mesentérica superior, com redução de 40%; aumento dos linfonodos retroperitoneais; e vesícula biliar com espessamento. O paciente foi, então, submetido a laparoscopia exploradora devido à refratariedade do quadro álgico. Durante o procedimento, não foram encontradas alterações que justificassem o quadro de dor apresentado pelo paciente, sendo descartadas todas as causas de abdome agudo possíveis. O paciente recebeu alta hospitalar precoce, com melhora do quadro.

Após os achados tomográficos da última internação, o paciente foi orientado a realizar angiotomografia de abdome no mês seguinte, cujo achado foi estenose do tronco celíaco (50%) (Figura 1). Após cerca de 6 meses, uma nova angiotomografia foi realizada, e só então foi notado espessamento do ligamento arqueado mediano do diafragma, com compressão e estenose de 80% do terço proximal do tronco celíaco.

**Figura 1:** Angiotomografia da aorta abdominal em reconstrução sagital da aorta e seus ramos. A seta demonstra estenose da porção proximal do tronco celíaco no pré-operatório e dilatação pós-estenótica.

O paciente foi submetido a cirurgia de descompressão do tronco celíaco por via laparoscópica. Durante a cirurgia, foi realizada dissecção da artéria hepática comum em direção ao tronco celíaco, local em que foram realizadas liberação das fibras do ligamento arqueado e retirada do gânglio celíaco adjacente. Durante a liberação do tronco celíaco, foi necessário realizar secção parcial das artérias esplênica e hepática comum para acessar as fibras do ligamento arqueado, pois tais vasos estavam intrinsecamente ligados a tais filamentos, o que ocasionou sangramento moderado, controlado mediante rafia primária das estruturas (Figura 2). O paciente recebeu hemotransfusão com um concentrado de hemácias no intraoperatório e realizou pós-operatório no centro de terapia intensiva, evoluindo com alta hospitalar após 3 dias.

**Figura 2:** Intraoperatório com demonstração do tronco celíaco após secção das fibras do ligamento arqueado. (A) Aorta abdominal; (B) Fibras do ligamento arqueado; (C) Artéria gástrica esquerda; (D) Artéria hepática comum com rafia primária; (E) Artéria esplênica com rafia primária.

O paciente manteve-se assintomático no pós-operatório e realizou angiotomografia de controle (Figura 3) 2 meses após a cirurgia, que evidenciou melhora da estenose do tronco celíaco entre 60-70%, além de estenose crítica de quase 100% do segmento proximal da artéria esplênica com fluxo distal através de circulação colateral originada da artéria gastroduodenal. Atualmente, após 1 ano da abordagem cirúrgica, o paciente mantém-se assintomático, e optou-se por não realizar mais exames de imagem devido à estabilidade do quadro.

**Figura 3:** Angiotomografia da aorta abdominal em reconstrução sagital da aorta e seus ramos. A seta demonstra melhora da estenose do tronco celíaco após abordagem cirúrgica.

## DISCUSSÃO

A SD foi identificada por Harjola em 1963, ao relatar o caso de um paciente com dor abdominal pós-prandial associada a sopro em região epigástrica^5^ . Após relatar uma série de casos em 1965, Dunbar descreveu e relacionou o conjunto de sinais e sintomas de dor abdominal pós-prandial, perda de peso, vômito, diarreia e sopro abdominal, à presença de compressão do tronco celíaco pelo ligamento arqueado mediano^3^ . A incidência da SD na população não é bem conhecida, mas a síndrome é mais comum em mulheres do que em homens (4:1), com idade entre 30 a 50 anos e em pacientes com biotipo físico magro^6^ .

A fisiopatogenia da SD não é completamente elucidada, sendo baseada em dois principais mecanismos: isquêmico e neuropático, podendo um ser predominante^7^ . O mecanismo isquêmico é justificado pela íntima relação existente entre o ligamento arqueado mediano e o tronco celíaco. O ligamento arqueado mediano é uma banda de tecido fibroso que conecta os pilares diafragmáticos direito e esquerdo, formando a margem anterior do hiato aórtico^3,6^ . Ambos os pilares diafragmáticos se originam dos corpos vertebrais da primeira à quarta vértebra lombar. Já o tronco celíaco se origina da aorta abdominal, com nível entre a décima primeira vértebra torácica e a primeira vértebra lombar. Dessa forma, indivíduos com origem mais alta do tronco celíaco ou inserção mais baixa do ligamento arqueado mediano apresentam maior predisposição para desenvolver compressão extrínseca do tronco celíaco, justificando o mecanismo isquêmico da SD^7^ .

Já o mecanismo neuropático é justificado pela presença do plexo celíaco ou gânglio celíaco. O plexo celíaco se encontra adjacente ao ligamento arqueado mediano e se origina dos nervos esplâncnicos pré-ganglionares, dos ramos somáticos dos nervos frênico e vago, dos nervos pré-ganglionares parassimpáticos e das fibras pós-ganglionares simpáticas^1,8^ . A dor associada à compressão do tronco celíaco pode ser mediada por esse plexo, pois pacientes submetidos a bloqueio com anestesia local do plexo celíaco apresentam melhora dos sintomas^8^ .

O diagnóstico da SD se inicia a partir da suspeita clínica em pacientes que apresentam os sinais e sintomas da síndrome; porém, deve ser confirmado com exames de imagem^9^ . Os exames radiológicos mais utilizados são angiografia, ultrassom com Doppler, tomografia/angiotomografia e ressonância/angiorressonância^9,10^ . A angiografia é considerada o padrão ouro para o diagnóstico da SD^5^ . Porém, com o desenvolvimento da angiotomografia *multislice*, é possível obter uma imagem bem acurada, com boa resolução e alto índice de detecção de lesões de forma bem menos invasiva que a arteriografia. Dessa forma, a angiotomografia é uma ótima escolha para o diagnóstico da SD^11^ .

Os achados arteriográficos de compressão do tronco celíaco, sobretudo em aquisições de projeção lateral, foram descritos como típicos, como estenose da porção proximal e deslocamento inferior do tronco celíaco em direção à aorta, seguido de inclinação cranial, assemelhando-se ao formato de U ou J, ou também denominado aspecto de gancho ou sinal do gancho^7^ . Em frequência variável, também podem ser observados aumento das artérias colaterais da arcada pancreaticoduodenal e dilatação pós-estenótica. A associação desses sinais e sintomas do trato gastrointestinal após exclusão de outras causas parecia justificar a descompressão cirúrgica do tronco celíaco^7,9^ .

Como precisávamos descartar causas mais comuns de dor abdominal neste caso, realizamos exames laboratoriais e TC de abdome, a qual evidenciou redução do calibre do tronco celíaco. Depois, realizamos angiotomografia, que evidenciou espessamento do ligamento arqueado mediano, com compressão do tronco celíaco. A SD é diagnosticada através da presença de sintomas gastrointestinais em associação com sinais radiológicos sugestivos, na ausência de outras causas que justifiquem os sintomas do paciente^7^ . Dessa forma, obtemos o diagnóstico de SD.

Em relação ao tratamento, embora a angioplastia com colocação de stent seja considerada uma boa opção para tratamento do componente aterosclerótico da obstrução do tronco celíaco, não apresentou bons resultados como tratamento único para a compressão da artéria celíaca, uma vez que a compressão extrínseca impede dilatação adequada do vaso pelo stent. Além disso, essa técnica é ineficaz no tratamento do componente nervoso da SD. A angioplastia possui um papel importante como procedimento secundário nos casos de estenose residual após descompressão do tronco celíaco^11^ .

O tratamento de escolha para a SD consiste em cirurgia para descompressão do tronco celíaco através da liberação do ligamento arqueado mediano e da retirada do gânglio celíaco, a fim de tratar ambos os componentes da doença. A cirurgia pode ser realizada por via aberta ou laparoscópica, sendo a laparoscopia a via de escolha por se tratar de um procedimento menos invasivo e com controle mais precoce dos sintomas. No entanto, ainda apresenta uma grande taxa de conversão aberta dada a alta morbidade associada ao sangramento agudo da aorta supracelíaca e de seus ramos^2,11^ . O refinamento das técnicas, a escolha de um cirurgião experiente e a identificação aprimorada de pacientes de alto risco pode diminuir a incidência de conversão^2^ . A abordagem isolada do componente isquêmico ou neurológico pode ser eficaz, uma vez que eles podem ocorrer de forma isolada^11,12^ . No presente caso, optou-se pela abordagem por videolaparoscopia, com descompressão do tronco celíaco utilizando tesoura coaguladora.

## CONCLUSÃO

A SD possui um diagnóstico ainda desafiador, porém, com o aprimoramento das técnicas radiográficas, a compressão do tronco celíaco vem sendo identificada com maior frequência, facilitando o diagnóstico. Estudos recentes vêm mostrando resultados satisfatórios com descompressão cirúrgica, seja por via aberta ou laparoscópica, sendo mais importante considerar a experiência do cirurgião com tais técnicas. Após a abordagem cirúrgica, é necessário realizar angiotomografia de controle para acompanhar a redução da estenose do tronco celíaco após a intervenção.
